# Diagnostic Value of CircRNAs as Potential Biomarkers in Oral Squamous Cell Carcinoma: a Meta-Analysis

**DOI:** 10.3389/fonc.2021.693284

**Published:** 2021-07-08

**Authors:** Mingfei Wang, Linfeng Zhang, Wenhao Ren, Shaoming Li, Keqian Zhi, Jingjing Zheng, Ling Gao

**Affiliations:** ^1^ School of Stomatology, Qingdao University, Qingdao, China; ^2^ Department of Oral and Maxillofacial Surgery, Key Laboratory of Oral Clinical Medicine, The Affiliated Hospital of Qingdao University, Qingdao, China; ^3^ Department of Endodontics, Key Laboratory of Oral Clinical Medicine, The Affiliated Hospital of Qingdao University, Qingdao, China

**Keywords:** circular RNA, OSCC, oral oncology, meta-analysis, biomarker, diagnosis

## Abstract

**Introduction:**

Circular RNAs (CircRNAs), an emerging non-coding RNA, have been demonstrated to be involved in tumorigenesis, metastasis, and cancer progression, and could represent novel potential biomarkers for diagnosing oral squamous cell carcinoma (OSCC). However, no meta-analysis has investigated the diagnostic role of circRNAs in OSCC. Hence, to investigate whether circRNAs could serve as specific biomarkers for OSCC, the present systematic review and meta-analysis evaluated the diagnostic efficiency of circRNAs in patients with OSCC.

**Materials and Methods:**

A thorough search of online databases (Pubmed, Web of Science, Embase, and the Cochrane Library) was conducted to collect relevant studies up to March 30th, 2021. All eligible studies were case-control studies. The quality of each study was evaluated by the Quality Assessment of Diagnostic Accuracy Studies-2 (QUADAS-2) tool. STATA (version 15.1) and Review Manager (version 5.4) were employed to conduct the meta-analysis, and the PRISMA statement was adopted in this study.

**Results:**

A total of 16 studies were included in the meta-analysis, with five studies on upregulated circRNAs, and 11 on downregulated circRNAs. The enrolled studies that met our eligibility criteria all derived from China. The pooled sensitivity (SEN), specificity (SPE), diagnostic odds ratio (DOR), positive likelihood ratio (PLR), negative likelihood ratio (NLR), and the area under receiver operating characteristics curve (AUC) with the 95% confidence intervals (95% CIs) were 0.74 (0.69–0.79), 0.79 (0.73–0.84), 10.74 (7.81–14.77), 3.50 (2.78–4.45), 0.33 (0.27–0.39) and 0.83 (0.79–0.86), respectively. The subgroup analysis demonstrated that serum, plasma, and saliva specimens had a better diagnostic performance than tissue samples, with a high value of sensitivity, specificity, DOR, and AUC values. The results also showed that the subgroups of upregulated circRNAs and a sample size of ≥100 manifested higher specificity, DOR, and AUC for cancer detection than downregulated circRNAs and a sample size of < 100.

**Conclusions:**

A strong association was demonstrated between the dysregulated expression of circRNAs and the diagnosis of OSCC. Hence, circRNAs have the potential to function as promising biomarkers and therapeutic targets for OSCC.

**Systematic Review Registration:**

PROSPERO, number CRD42021256857.

## Introduction

Head-and-neck squamous cell carcinoma (HNSCC) ranks the sixth most common neoplasm by incidence globally, accounting for 650,000 new cancer cases and 350,000 deaths worldwide annually (1). HNSCCs constitute a group of epithelial malignant tumors in the oral cavity, nose, sinuses, salivary gland, larynx, and pharynx. According to previous reports, males are more likely to be affected than females, with a ratio ranging from 2:1 to 4:1 (2). Among the subtypes of HNSCC, oral squamous cell carcinoma (OSCC) is the most common malignant neoplasm and has a poor prognosis with a 5-year survival rate of <50% (3, 4). The most widely applied therapies include surgery, chemotherapy, and radiotherapy, which significantly compromise the patients’ quality of life. In etiology, the typical risk factors are mainly related to environmental carcinogens, such as tobacco and alcohol, and risky lifestyle habits, e.g., betel nut chewing (5). Recently, the human papillomavirus (HPV) has emerged as an etiologic factor contributing to the development of HNSCC (6). HPV-positive HNSCC cases, as a consequence of HPV infections, mainly occur in the oropharynx region within the lymphoid epithelium of the tongue or tonsils, primarily in patients with the HPV-16 subtype (7).

Currently, the gold standard for OSCC diagnosis is still conventional oral examination and the histological evaluation of biopsy tissue, constituting highly accurate and reliable diagnostic methods with high specificity and sensitivity. However, the clinical application is limited due to patient discomfort and sampling bias, leading to misdiagnosis (8). Some biomarkers (such as carcinoembryonic antigen [CEA] and CA199) have been developed and implemented clinically. However, they have low accuracy and have proven inefficient. Consequently, it is imperative for clinicians to search for novel biomarkers as non-invasive diagnostic tools to enhance the efficacy of OSCC diagnosis.

Circular RNAs (circRNAs), a novel class of endogenous non-coding RNAs, are derived from the back-splicing by the canonical spliceosome *via* exon or intron circularization (9). As the high-throughput sequencing technology has made great strides and been widely employed, several circRNAs have been captured and identified (10). Instead of the linear structure within a 5’ cap and a 3’ polyadenylated tail, this non-coding RNA is characterized by a covalently closed-loop structure (11). CircRNAs are exceedingly stable and play a pivotal role in various physiological and pathophysiological processes. Studies recently published implied that this newly found subclass of long non-coding RNA has significantly boosted research efforts in many diseases, such as heart failure, autism, diabetes mellitus, and cancer (12–15). Cumulative research has illustrated that circRNAs function as microRNA molecular sponges and regulate gene expression and other biological procedures, such as cell proliferation, invasion, and migration (16).

Considerable evidence has indicated that circRNAs could serve as a viable diagnostic option. Nonetheless, due to variations in the study design, specimen type, and sample size, no explicit clinical diagnostic significance of circRNA in OSCC has been elucidated in previous studies. Therefore, this systematic meta-analysis aimed to combine the results of previously published studies to estimate the diagnostic test accuracy of dysregulated circRNAs as biomarkers for OSCC.

## Materials and Methods

The process of study selection was conducted following the Preferred Reporting Items for Systematic Reviews and Meta-Analyses guideline for diagnostic test accuracy (PRISMA-DTA) (17).

### Study Design

A systematic review and meta-analysis were applied to assess the clinical diagnostic capability of circRNAs in OSCC.

### Bibliography Search Strategy

All the eligible studies in this meta-analysis were selected independently by two authors (MW and LZ). A thorough electronic search was carried out in PubMed, Web of Science, Embase, and Cochrane Library online databases up to March 30th, 2021. The full and reproducible keywords used in the search are provided in **Appendix 1**. In addition, the two authors independently and manually screened the titles, abstracts, and full texts to identify the relevant studies. Then the authors extracted the data from the relevant articles.

### Inclusion and Exclusion Criteria

All the studies included in the meta-analysis met the following inclusion criteria.

The inclusion criteria were as follows:

- case-control study or cohort study,
- the diagnosis of oral squamous cell carcinoma was confirmed by histological examinations,
- the studies analyzed the relationship between circRNAs and oral cancers,
- circRNAs expression levels were assessed with quantitative real-time polymerase chain reaction(qRT-PCR) analysis, and
- the sample size, sensitivity, specificity, and AUC were provided to calculate true positives (TP), false positives (FP), false negatives (FN), and true negatives (TN);

The exclusion criteria were as follows:

- duplicate data from previous studies,
- reviews, letters, case reports, and meeting abstracts, etc.,
- non-English and animal studies, and
- insufficient or unqualified data.

### Data Extraction and Quality Assessment

The titles and abstracts of the articles were independently screened by two authors (MW and LZ) to determine their relevance to the topic, focusing on the diagnostic application of RNA in OSCC, defined by the International Classification of Diseases 10th Revision (ICD-10) codes C00-C06. Then LG and JZ evaluated the full text of the remaining articles, independently extracted the relevant data, and cross-checked to ensure data accuracy. The following data were extracted from each study: (a) basic information including the first author’s name, publication year, country, circRNA type, circRNA expression, sample size, cancer type, specimen, and detection method; (b) clinicopathological features including gender, age, tumor size, lymph node metastasis, TNM, T-stage, differentiation, and extrathyroidal extension; and (c) diagnostic information including sample size, sensitivity, specificity, and area under the curve (AUC).

The quality of each study was assessed independently by two authors (WR and SL) using the Quality Assessment of Diagnostic Accuracy Studies-2 (QUADAS-2) tool, retaining all original domain questions in two dimensions (“Risk of Bias” and “Applicability Concerns”) (18). Each risk of bias item was graded “yes”, “no”, or “unclear”, while the applicability concerns were evaluated as “high”, “low”, or “unclear”.

### Summary Measures

The diagnostic sensitivity and specificity of the upregulated or downregulated circRNAs in the OSCC patients compared to the controls were considered the primary measures.

### Statistical Analysis

The meta-analysis was conducted utilizing STATA 15.1 and Review Manager 5.4 statistical softwares to analyze the diagnostic performance of circRNAs in OSCC, constructing forest plots for sensitivity (SEN), specificity (SPE), negative likelihood ratio (NLR), positive likelihood ratio (PLR), and the diagnostic odds ratio (DOR). A summary receiver operator characteristics curve (SROC) was plotted to calculate the area under the SROC curve (AUC) and 95% confidence intervals (95% CIs) for the qualitative assessment of the diagnostic value. Deek’s funnel plot and funnel chart were constructed to estimate the publication bias between the included studies (with P > 0.05 indicating no publication bias). Furthermore, the Harbord test plot was established to scrutinize the potential publication bias in the meta-analysis (with P > 0.05 indicating no publication bias). Fagan’s nomogram was constructed to calculate post-test probabilities. An LR scatter matrix plot was utilized to assess the clinical significance of individual diagnostic studies, which was divided into four quadrants. Heterogeneity was estimated using I^2^ statistics and the Cochrane Q-test (with I² > 50% and P < 0.05 suggesting significant heterogeneity). The analysis applied a random-effects model due to significant heterogeneity. Meta-regression and subgroup analyses were performed to identify the potential source of heterogeneity. Sensitivity analysis was conducted by omitting individual studies to test the reliability of our analyses.

### Trial Sequential Analysis

Trial sequential analysis (TSA) was performed for the meta-analysis results using the TSA software V.0.9.5.5 beta (Copenhagen Trial Unit). This analysis was utilized to estimate the required information size (RIS) for the statistical significance of the present meta-analysis. When the actual sample size in the meta-analysis failed to reach the RIS, TSA was applied to combine the results and provide a cumulated sample size of the included studies with an adjusted threshold to test the statistical significance and considerably reduce type I errors (false-positive results). Theoretically, the cumulative z-curve crossing both the conventional and TSA monitoring boundaries indicated sufficient evidence for the diagnostic capability of dysregulated circRNAs for OSCC detection. The required information size, adopting an alpha risk of 5% and a beta risk of 20%, was estimated for this analysis.

## Results

### Search Results

**Figure 1:**
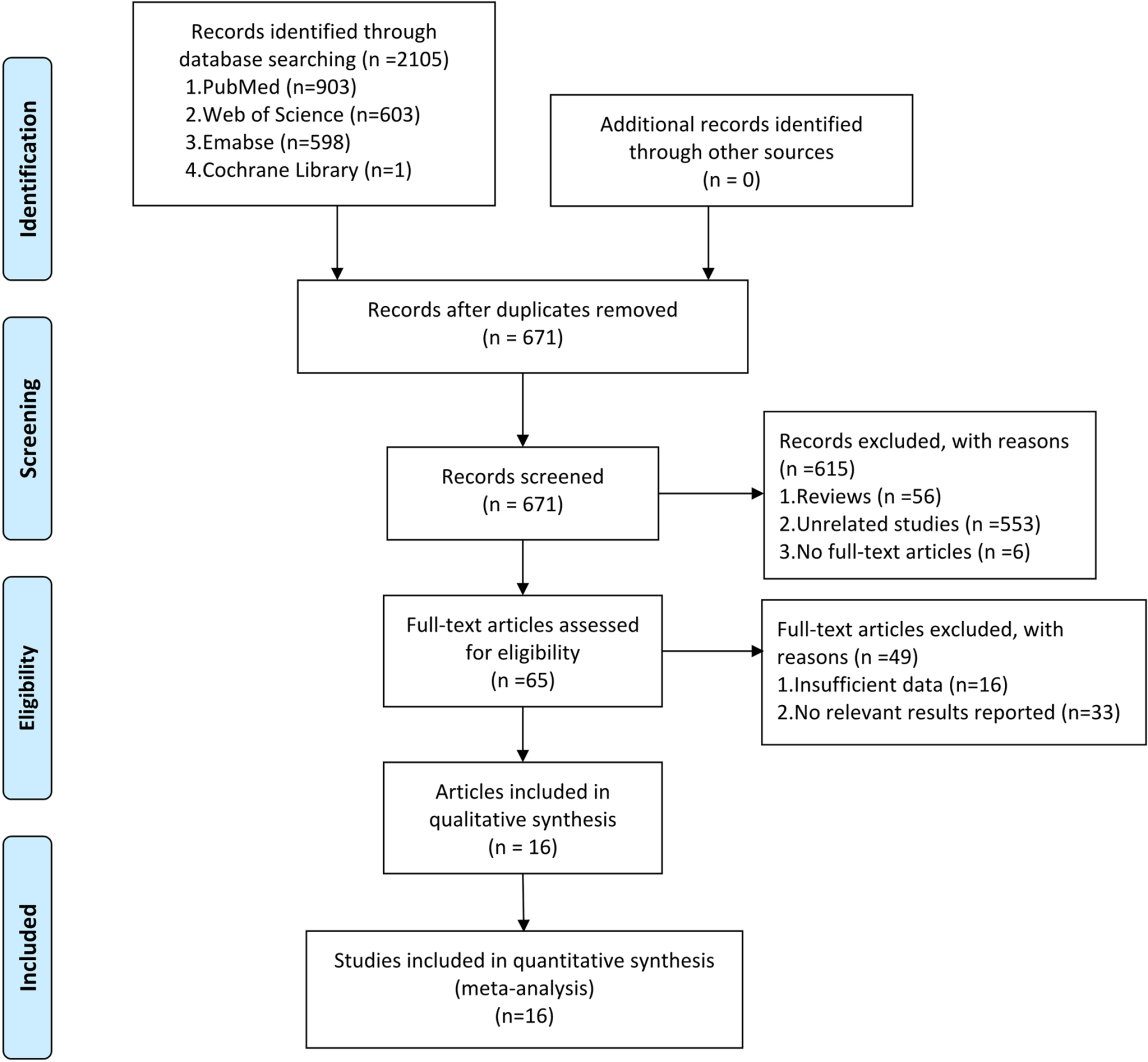
The PRISMA flow diagram showing the process of eligible studies search and selection. This meta-analysis identified 16 eligible studies that used circRNAs as biomarkers for tumor diagnosis.

**Figure 1** presents the detailed search process. A total of 2105 potentially eligible articles were identified from PubMed (903 records), Web of Science (603 records), Embase (598 records), and Cochrane Library (one record). A total of 671 records remained after eliminating 1434 duplicates. Furthermore, 615 articles were excluded for the following reasons: 56 were review articles, 553 were unrelated studies, and six were articles whose full texts were not available. After evaluating the full articles, 33 records were excluded without sufficient data, and 16 were removed because no relevant results were reported (**Appendix 2**). Finally, 872 cases and 900 controls from 16 studies were included in the meta-analysis (19–34).

### Study Characteristics

In the meta-analysis, an evaluation was conducted on the association between circRNA expression levels and the type of OSCC to determine the accuracy of circRNA expression as an OSCC biomarker. **Table 1** presents the main characteristics of each study. All the studies were released between 2018 and 2021, which were all from China. Each included study adopted the conventional case-control design. Twelve studies used paired OSCC as cases and corresponding adjacent normal tissue as controls. Four studies used saliva, plasma, or serum samples from OSCC patients as the cases and healthy volunteers as controls. The number of patients in the included studies ranged from 25 to 146. A total of 16 different circRNAs were assessed, among which five upregulated circRNAs were recognized as tumor promoters (22, 25, 29, 30, 32) and 11 were downregulated as tumor suppressors (19–21, 23, 24, 26–28, 31, 33, 34). All circRNA expression levels were detected using qRT-PCR in tissues (n = 12), plasma (n = 1), serum (n = 2), and saliva (n = 1). All the samples were collected before clinical treatment.

**Table 1:** Main characteristics of 16 studies included in the meta-analysis.

	First author	Year	Country	CircRNA	Regulation	Sample size		Cancer type	Specimen	Method	Diagnostic power			Source of the control group
						Case	Control				Sensitivity	Specificity	AUC	
1	Sun S	2018	China	hsa_circ_001242	downregulated	40	40	OSCC	tissue	qRT-PCR	0.725	0.775	0.784	adjacent normal tissues
2	Li B	2018	China	hsa_circ_0008309	downregulated	45	45	OSCC	tissue	qRT-PCR	0.512	0.913	0.7642	adjacent normal tissues
3	He T	2018	China	circPVT1	upregulated	50	50	OSCC	tissue	qRT-PCR	0.686	0.86	0.787	adjacent normal tissues
4	Zhao S	2018	China	hsa_circ_0001874+ hsa_circ_0001971	upregulated	93	85	OSCC	saliva	qRT-PCR	0.9268	0.7778	0.922	Healthy controls
5	Li X	2019	China	hsa_circ_0004491	downregulated	40	40	OSCC	tissue	qRT-PCR	0.73	0.68	0.751	adjacent normal tissues
6	Xia B	2019	China	circ-MMP9	upregulated	25	16	OSCC	plasma	qRT-PCR	0.889	0.81	0.91	Healthy controls
7	Su W	2019	China	hsa_circ_0005379	downregulated	37	37	OSCC	tissue	qRT-PCR	0.699	0.605	0.6805	adjacent normal tissues
8	Dou Z	2019	China	hsa_circ_0072387	downregulated	63	63	OSCC	tissue	qRT-PCR	0.714	0.698	0.746	adjacent normal tissues
9	Fan C	2019	China	circMAN1A2	upregulated	55	121	OSCC	serum	qRT-PCR	0.672	0.915	0.779	Healthy controls
10	Wang Z	2019	China	hsa_circ_009755	downregulated	27	27	OSCC	tissue	qRT-PCR	0.7037	0.7778	0.782	adjacent normal tissues
11	Yao Y	2020	China	circular RNA_0001742	upregulated	146	146	OSCC	tissue	qRT-PCR	0.775	0.808	0.87	adjacent normal tissues
12	Zhang H	2020	China	hsa_circ_0003829	downregulated	60	60	OSCC	tissue	qRT-PCR	0.7	0.8	0.81	adjacent normal tissues
13	Li L	2020	China	hsa_circ_0086414	downregulated	55	55	OSCC	tissue	qRT-PCR	0.655	0.873	0.749	adjacent normal tissues
14	Chen G	2020	China	circATRNL1	downregulated	48	48	OSCC	tissue	qRT-PCR	0.848	0.509	0.711	adjacent normal tissues
15	Zhang B	2020	China	hsa_circ_009755	downregulated	42	42	OSCC	tissue	qRT-PCR	0.69	0.885	0.83	adjacent normal tissues
16	Fan X	2021	China	circSPATA6	downregulated	46	25	OSCC	serum	qRT-PCR	0.79	0.69	0.7748	Healthy controls

### Risk of Bias and Applicability Concerns Within Studies

**Figure 2:**
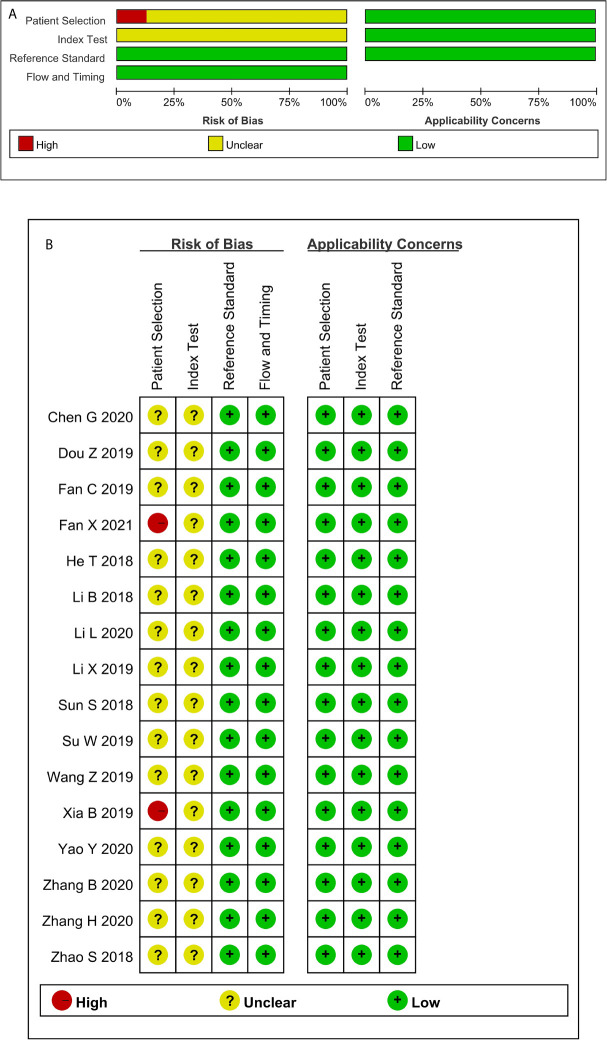
Quality assessment of the included studies according to QUADAS-2 **(A)** Methodological quality graph; **(B)** Methodological quality summary.

Not a single included study fulfilled all the domain criteria in the QUADAS-2 methodological quality tool. On average two out of four domains of risk of bias were fulfilled in each study. The case-control design and inappropriate exclusions(for the specific diagnosis) explained why no study was observed to have a low risk in patient selection and index test domain. Two of these studies were graded as high risk in patient selection because no exact time scope and continuity were mentioned. Items 4 and 7 were assessed as unclear because no information on blinding was reported. All the articles met the criteria of the three domains of applicability concerns (**Figure 2** and **Appendix 3**).

### Meta-Analysis

**Figure 3:**
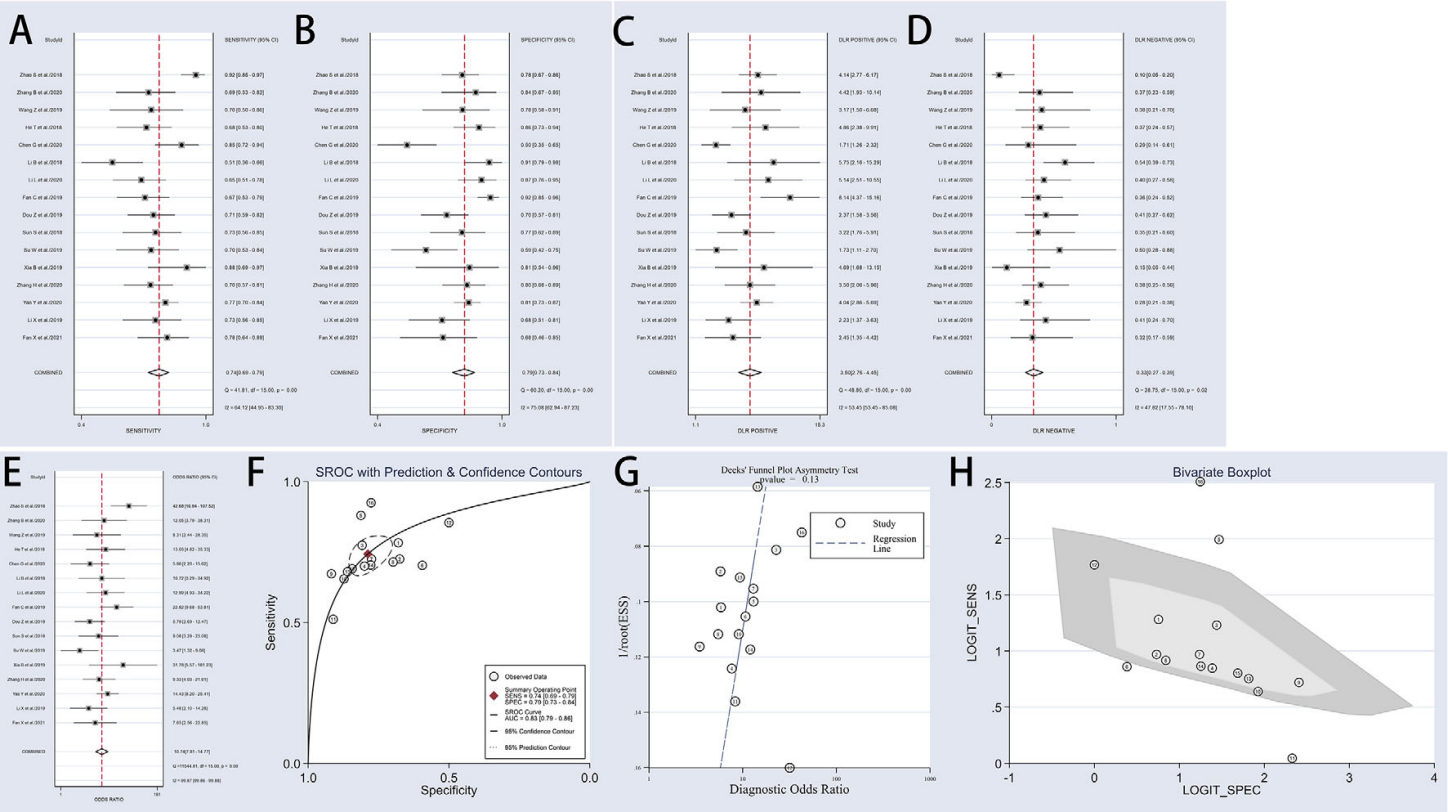
Forest plots of sensitivity, specificity, PLR, NLR, DOR, AUC, and funnel plot for diagnosis of circRNAs in OSCC among 16 studies. **(A)** Sensitivity; **(B)** Specificity; **(C)** PLR; **(D)** NLR; **(E)** DOR; **(F)** AUC (SROC curve); **(G)** Deek’s funnel plot; and **(H)** Bivariate boxplot. OSCC, oral squamous cell carcinoma; PLR, positive likelihood ratio;NLR, negative likelihood ratio; DOR, diagnostics odds ratio; SROC, Summary receiver operator characteristics curve; AUC, the area under SROC curve.

**Figure 4:**
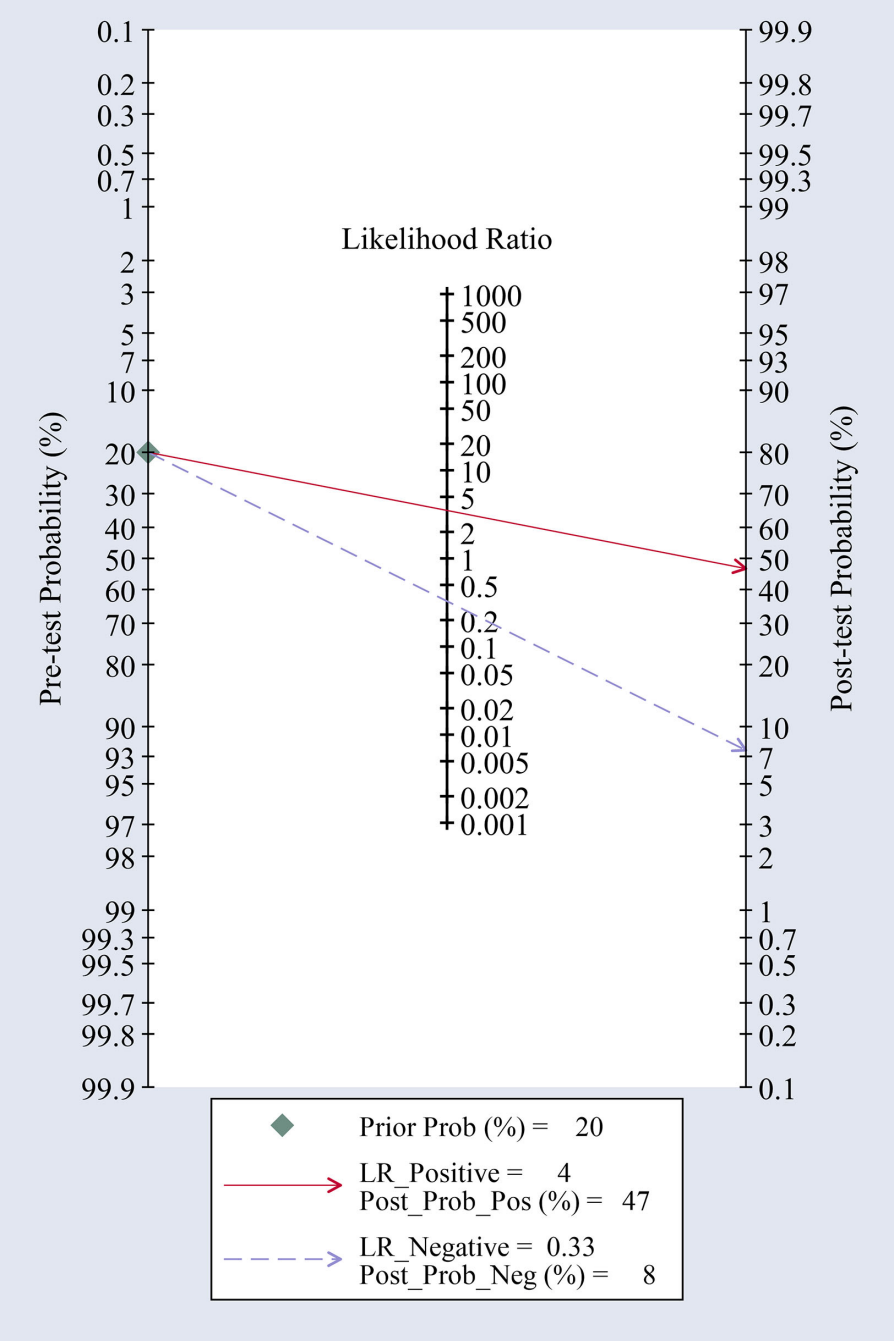
Fagan’s nomogram for likelihood ratios.

**Figure 5:**
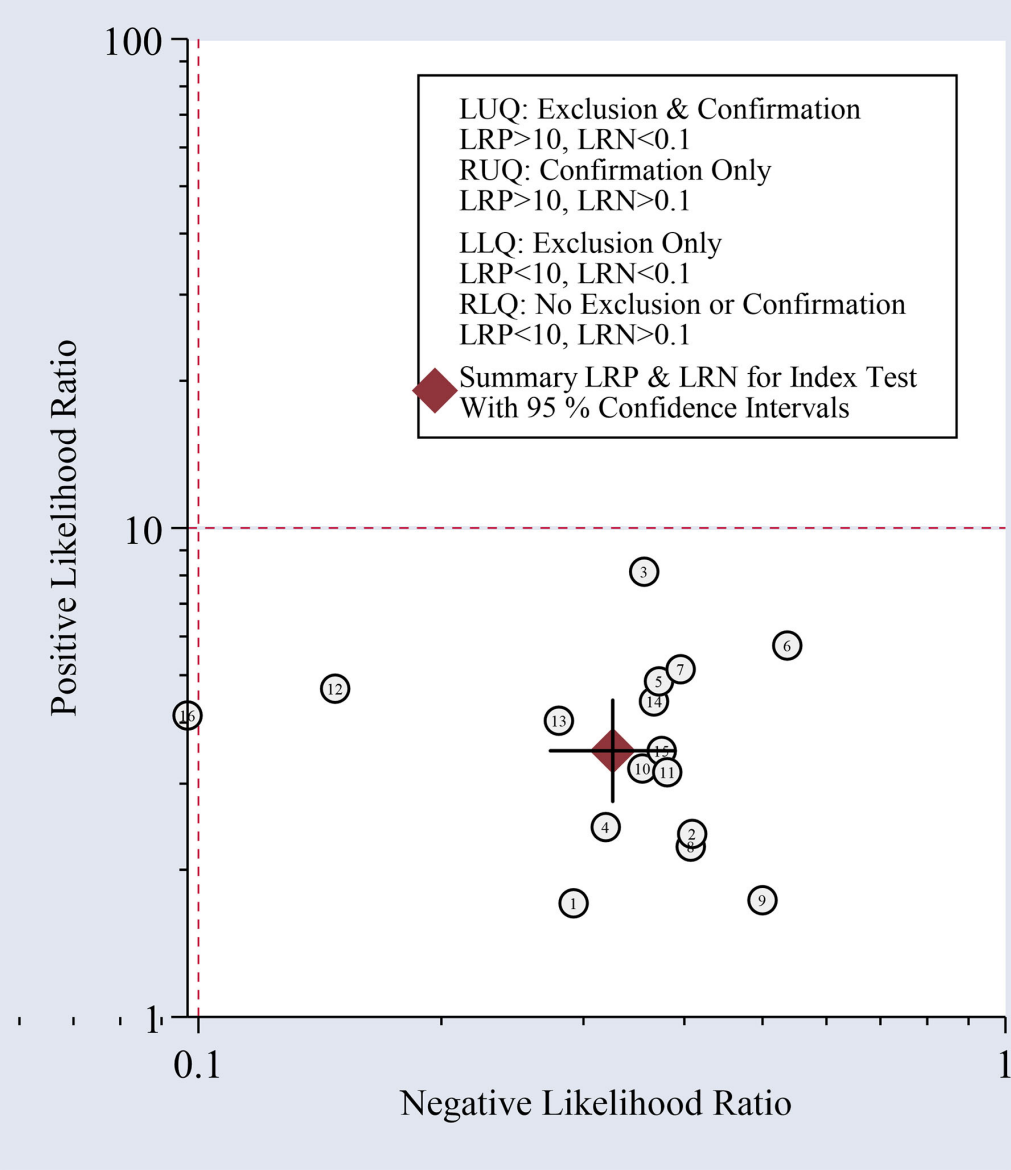
Scatter plot of positive and negative likelihood ratios with combined summary points.

The present meta-analysis of 16 cohorts in 872 patients and 900 controls included 16 circRNA types. A random-effects model was selected because of the significant heterogeneity (I² > 50%) between the included studies. The meta-analysis was conducted, and the pooled sensitivity, specificity, PLR, NLR, DOR, and SROC were calculated for circRNA, as illustrated in **Figure 3**. The pooled statistical values for sensitivity (**Figure 3A**), specificity (**Figure 3B**), PLR (**Figure 3C**), NLR (**Figure 3D**), and DOR (**Figure 3E**) with the 95% confidence intervals for the enrolled studies in this study were 0.74 (95% CI: 0.69 – 0.79), 0.79 (95% CI: 0.73 – 0.84), 3.50 (95% CI: 2.76 – 4.45), 0.33 (95% CI: 0.27 – 0.39), and 10.74 (95% CI: 7.81 – 14.77), respectively. The diagnostic odds ratio (DOR) represents a critical indicator that assists in a meta-analysis by focusing on diagnostic performance, and combines the advantages of both sensitivity and specificity, and describes the diagnostic value of a circRNA (35). The summary receiver operator curve (SROC, **Figure 3F**) plot revealed an AUC of 0.83 (95% CI: 0.79 – 0.86). The bivariate boxplot in **Figure 3H** presents the heterogeneity details in the included studies. Fagan’s nomogram was constructed to calculate the post-test probabilities of the circRNAs, in which the post-test possibility increased to 47% with a positive likelihood ratio (LR) of 4, with the post-test possibility decreasing to 8% with a negative LR of 0.33 (**Figure 4**). These findings indicated that circRNAs were a credible diagnostic biomarker with high accuracy and efficacy. **Figure 5** presents an LR scattergram plotted with the combined summary points.

Therefore, taken together, the results indicated that the circRNAs had good diagnostic accuracy for OSCC and could serve as effective biomarkers of OSCC.

### Meta-Regression and Subgroup Analysis

Overall, the studies exhibited relatively high heterogeneity in sensitivity and specificity (I² = 64.12 and I² = 75.08, respectively). Thus, a meta-regression analysis was first conducted to investigate the heterogeneity source (**Appendix 4**). The meta-regression analysis demonstrated that three covariates (specimen type, expression status, and sample size) could explain 100% of the between-study variance.

**Figure 6:**
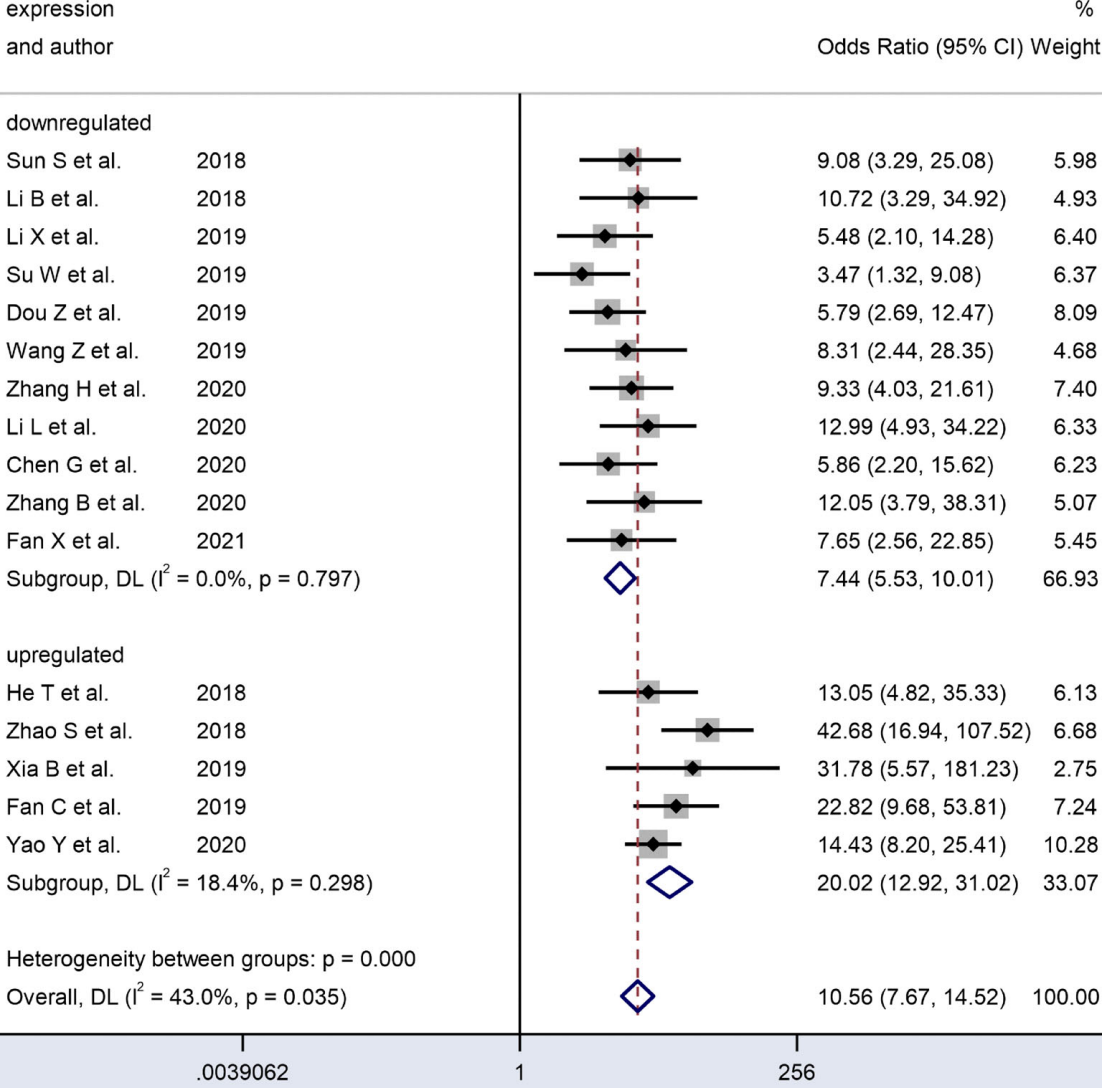
Forest plots of subgroup analysis of the combined ORs with 95%CIs according to expression status of circRNAs in patients with OSCC.

**Figure 7:**
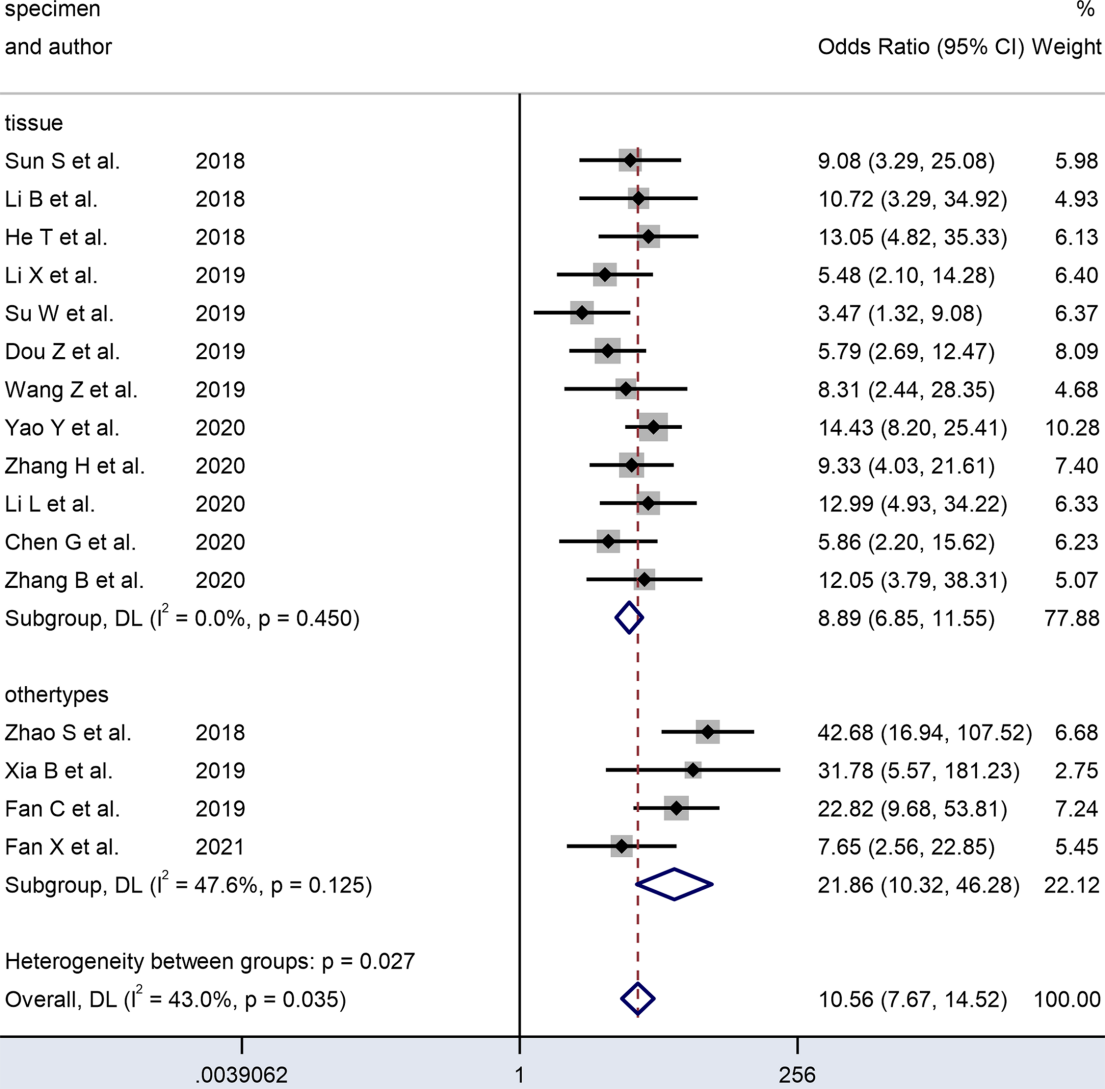
Forest plots of subgroup analysis of the combined ORs with 95%CIs according to specimen type of circRNAs in patients with OSCC.

**Figure 8:**
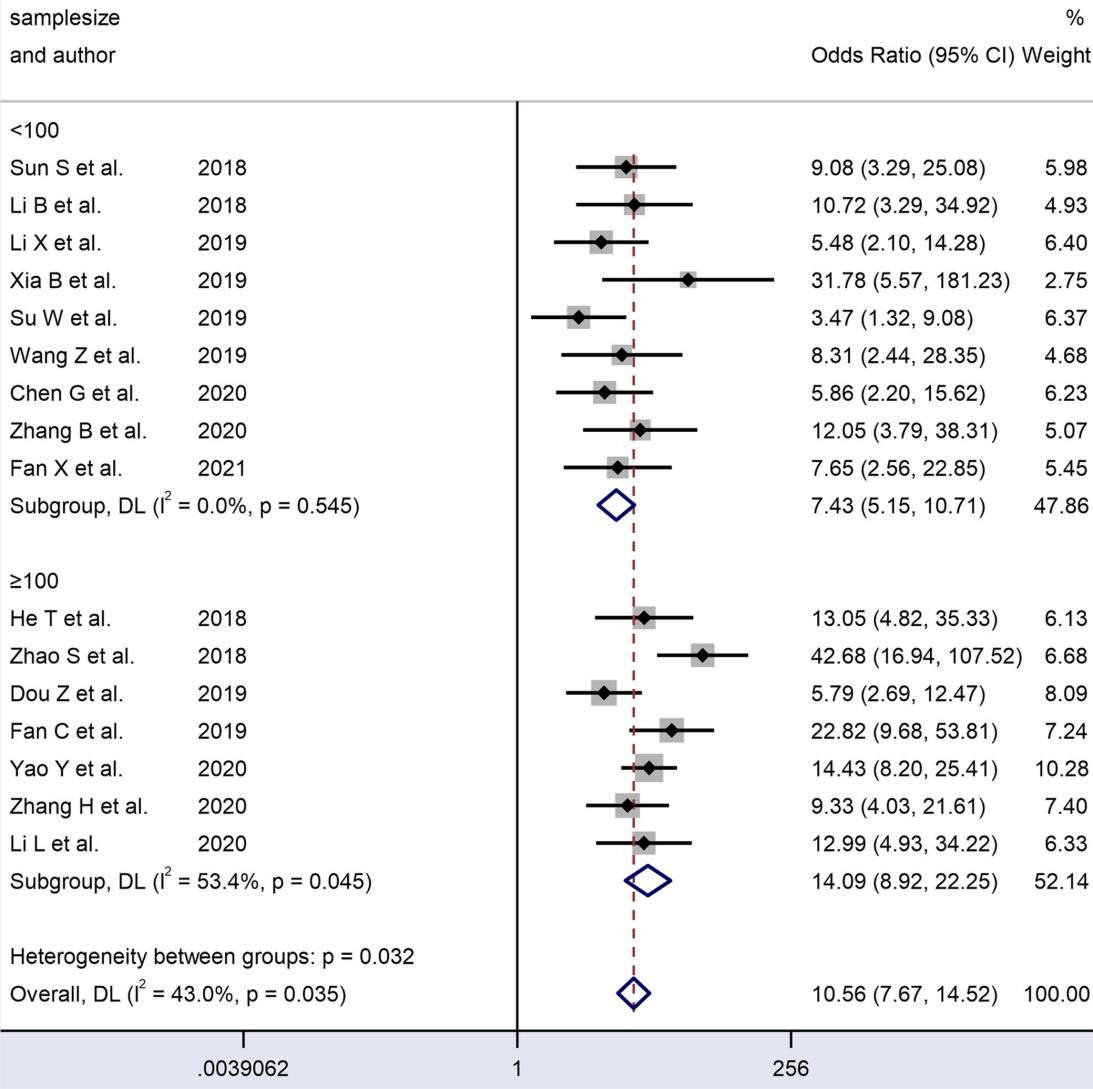
Forest plots of subgroup analysis of the combined ORs with 95%CIs according to sample size of circRNAs in patients with OSCC.

**Table 2** and **Figures 6**–**8** present further subgroup analyses to identify the source of the heterogeneity. Subgroups of studies utilizing serum (n = 2), plasma (n = 1), and saliva (n = 1) specimens exhibited better diagnostic performance with DOR (24.70 *vs.* 9.00) and the AUC (0.90 *vs.* 0.80) compared to the tissue (n = 12) subgroup. The pooled sensitivity and specificity were both greater than in the tissue subgroup. No significant heterogeneity was observed in the tissue subgroup (I² = 0.0, P = 0.450) or other specimen types (I² = 47.6%, P = 0.125). Therefore, the variance between these two subgroups of specimen types may account for the heterogeneity.

**Table 2:** Results of subgroup analysis of cricRNAs reported by 16 studies in diagnostic meta-analysis.

Analysis	No. of studies	Sensitivity (95% CI)	Specificity (95% CI)	PLR (95% CI)	NLR (95% CI)	DOR (95% CI)	AUC(95%CI)	I²(%)	p-value
Overall	16	0.74(0.69-0.79)	0.79(0.73-0.84)	3.50(2.78-4.45)	0.33(0.27-0.39)	10.74(7.81-14.77)	0.83(0.79-0.86)	43.0	0.035
Sample type									
tissue	12	0.72(0.7-0.76)	0.78(0.71-0.84)	3.26(2.50-4.24)	0.36(0.32-0.42)	9.00(6.61-12.25)	0.80(0.76-0.83)	0.0	0.450
serum or plasma or saliva	4	0.83(0.71-0.91)	0.83(0.74-0.90)	4.93(3.32-7.33)	0.20(0.12-0.34)	24.70(14.37-42.44)	0.90(0.87-0.92)	47.6	0.125
Sample size									
≥100	7	0.75(0.66-0.82)	0.83(0.77-0.87)	4.36(3.33-5.70)	0.31(0.23-0.41)	14.22(9.41-24.50)	0.86(0.83-0.89)	53.4	0.045
<100	9	0.73(0.66-0.80)	0.73(0.64-0.81)	2.76(2.06-3.71)	0.36(0.30-0.45)	7.58(5.17-11.11)	0.79(0.76-0.83)	0.0	0.545
Expression									
downregulated	11	0.71(0.65-0.76)	0.75(0.67-0.82)	2.88(2.20-3.76)	0.38(0.33-0.45)	7.49(5.38-10.44)	0.78(0.74-0.82)	0.0	0.797
upregulated	5	0.80(0.68-0.88)	0.84(0.78-0.89)	4.94(3.75-6.50)	0.24(0.16-0.38)	20.35(13.10-31.62)	0.89(0.86-0.91)	18.4	0.298

We analyzed the subgroups according to the expression status of dysregulated circRNAs. The studies on upregulated circRNAs (n = 5) had a significantly higher pooled DOR (20.35 *vs.* 7.49) and AUC (0.89 *vs.* 0.78) than those on downregulated circRNAs (n=11). In the forest plots, the results covered no heterogeneity in upregulated (I² = 18.4%, P = 0.298) and downregulated circRNAs (I² = 0.0, P = 0.797). Since no heterogeneity was observed in the subgroups, the difference between these two expression status subgroups may account for the heterogeneity.

An analysis of subgroups classified by the sample size of the included cohorts (≥ 100 and < 100) was also carried out. The ≥ 100 subgroup (n = 7) had a higher DOR (14.22 *vs.* 7.58) and AUC (0.86 *vs.* 0.79) than the < 100 subgroup (n = 9) (**Table 2**). In the former subgroup, evident heterogeneity was detected with a value of I² = 53.4 (P = 0.045). However, no heterogeneity was observed in the latter subgroup (I² = 0.0, P= 0.545). Hence, the subgroup analysis results indicated that the sample size of the enrolled cohorts might be the source of heterogeneity.

### Publication Bias

**Figure 9:**
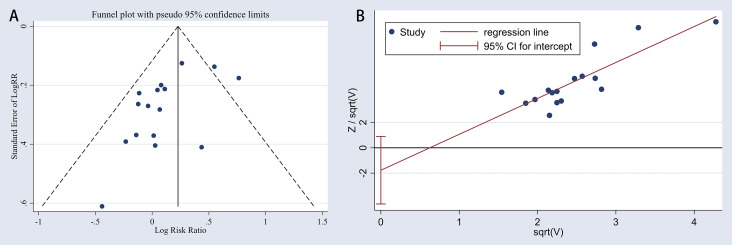
Publication bias in the meta-analysis. **(A)**, funnel chart; **(B)**, Harbord test plot.

Deek’s funnel plot asymmetry tests were employed to assess the publication bias, as illustrated in **Figure 3G**, with the results indicating no obvious publication bias (P = 0.13). The funnel plot and Harbord test shown in **Figure 9** were used to track the potential publication bias in the meta-analysis. The P-value of both was > 0.05 (P = 0.13 and P = 0.175), suggesting no publication bias in the meta-analysis.

### Sensitivity Analysis

**Figure 10:**
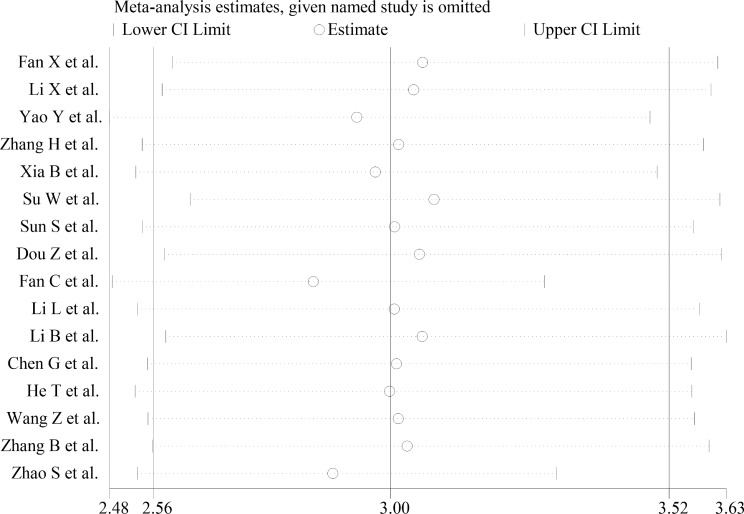
Sensitivity analysis plot of overall included studies.

A sensitivity analysis was carried out to explain the heterogeneity of each study. As shown in **Figure 10**, omitting any individual study had no substantial impact on the pooled statistics, indicating that the results were credible and reliable.

### TSA Outcome

The overall required information size was calculated for the 1762 participants (**Appendix 5**). The z-curve crossed both the conventional and TSA monitoring boundaries. However, it failed to reach the RIS line, indicating statistical significance and sufficient evidence on the diagnostic performance of circRNAs as biomarkers for OSCC.

## Discussion

Previous cumulative investigations have demonstrated that dysregulated circRNAs played a critical role in the cell proliferation, metastasis, and occurrence of various cancers. The closed, covalent, and continuous circular structure of circRNAs makes them more steady than their linear counterparts (36). Moreover, dysregulated circRNAs have been discovered in plasma, tissues, and serum (37). The characteristics above render circRNAs favorable as molecular biomarkers of cancer.

Overall, 16 studies were included in this meta-analysis. The final results based on all the enrolled studies showed an AUC of 0.83 for circRNAs, with a sensitivity of 0.74 and specificity of 0.79 in distinguishing OSCC patients from healthy controls. The pooled sensitivity and specificity showed moderate diagnostic test accuracy, indicating that circRNAs had sufficient statistical ability to identify or exclude suspected cases to enhance the clinical diagnosis. A DOR of 10.74 (> 1.0) was obtained in the present analysis, indicating that the dysregulated circRNAs were effective predictive biomarkers for OSCC. Furthermore, the higher AUC value showed better performance in the balance of sensitivity and specificity. Notably, the high AUC value of 0.83 in the current analysis reflects the overall relatively high diagnostic accuracy of circRNAs in OSCC detection.

To the best of our knowledge, this study was the first systematic review and meta-analysis to estimate the diagnostic ability of circRNAs as biomarkers in OSCC patients and summarize their sensitivity and specificity. Other published systematic reviews have evaluated several biomarkers for OSCC diagnosis, such as microRNAs, mRNAs, and proteins. Rapado-Gonzalez et al. performed a systematic review of microRNAs in OSCC and summarized the clinical correlation, including proliferation and progression, with a relatively high AUC of 0.91 (38). Gaba et al. assessed the diagnostic value of a specific mRNA, DUSP1, which proved insufficient with an AUC of 0.66. In addition, Gaba et al. reviewed the clinical correlation of a protein named IL1-β protein and estimated its diagnostic value, which was considered good with an AUC of 0.82 (39). However, despite the growing number of reviews and analyses based on diagnostic biomarkers for OSCC, no consensus has been reached on determining which biomarker has the best diagnostic performance for OSCC and what is the most accurate sample for testing.

Meta-regression and subgroup analyses of specimens, sample size, and the expression status of the dysregulated circRNAs were performed to explore the sources of the heterogeneity. The random-effects-based meta-regression analysis showed that these three covariates were the main sources of the heterogeneity. Studies that utilized serum, plasma, and saliva specimens performed significantly better in diagnosing OSCC patients compared to those using tissue specimens, with no heterogeneity detected. The subgroups with upregulated circRNAs and ≥ 100 samples were found to possess a much higher diagnostic accuracy than the downregulated circRNAs and those with < 100 samples and these two subgroups showed no heterogeneity. Apparent heterogeneity was found in the subgroup with ≥ 100 samples. Therefore, the heterogeneity might be related to the sample size, specimen type, and expression status.

We further considered the reasons for the differences in the diagnostic accuracy between the tissue and liquid biopsies. Concerning tissue biopsies, OSCC, as a solid tumor, exhibits tissue heterogeneity even for the same histological type. The proportion of tumor cells and mesenchymal cells vary in different patients and even in different parts of the same tumor. Therefore, the sample used for detection only accounts for part of the tumor. This cannot accurately reflect the whole tumor status, which is the potential reason for the lower accuracy in tissue samples than detection by body fluid specimens.

Currently, tissue-based diagnostic strategies require the testing of materials obtained through invasive procedures, such as biopsy or aspiration, which are usually associated with severe discomfort and medical costs (40). Compared to tissue biopsies, body fluids are a better choice for disease screening and diagnosis due to the advantages of accessibility, low invasion, low cost, and various sample types to monitor disease development (41). Several studies have reviewed the potential of mRNAs as diagnostic markers, illustrating the possible clinical application of OSCC-specific signals in body fluids (42, 43). These molecules can be prospective candidates for biomarkers due to their stable circulation in human body fluids and accessibility through non-invasive methods. Likewise, circRNA detection can be regarded as a novel method for body fluid-based biopsies, which would be helpful as significant diagnostic and monitoring tools in the clinic. At present, there is a shortage of research on the diagnostic value of circRNAs in body fluids for OSCC, which provides a new direction for researchers worldwide to utilize the saliva and serum of high-risk patients with lesions in the oral cavity suspected of oral squamous cell carcinoma.

Nevertheless, it is not yet possible to apply these molecular biomarkers in clinical diagnosis and monitoring. New techniques, such as digital PCR, make it possible to test cancer and phenotype-specific molecular changes to improve sensitivity and accuracy. After this technique is used more widely, the successful application of molecular markers in liquid biopsies of other tumors (e.g., lung carcinoma) will encourage further evaluation of this method in OSCC cases.

Several deficiencies in the present study merit consideration. (a) Based on our inclusion criteria, all samples and relevant statistics accidentally came from China. (b) Subgroup analysis of different circRNA should be further performed. (c) Conspicuous heterogeneity existed in the included studies. The sample size, specimen types, and expression status might be sources of the heterogeneity. (d) The sample size and number of the enrolled studies in this analysis were relatively small. Thus, more comprehensive high-quality studies that encompass larger scales, more regions, well-designed operations, and further exploration into the functional mechanisms are necessary.

## Conclusion

In summary, this meta-analysis revealed a strong association between the altered expression of circRNAs and the diagnosis of OSCC. Hence, circRNAs can potentially serve as promising biomarkers and therapeutic targets for OSCC. Nevertheless, the clinical application of cricRNAs for the detection of OSCC requires further research.

## Data Availability Statement

The original contributions presented in the study are included in the article/**Supplementary Material**. Further inquiries can be directed to the corresponding authors.

## Author Contributions

LZ contributed to conception, analysis, and drafted manuscript. MW contributed to conception and design, acquisition and interpretation of data, and critically revised manuscript. WR contributed to design, interpretation of data, and drafted manuscript. SL contributed to design, acquisition of data, and critically revised manuscript. KZ critically revised manuscript. JZ contributed to acquisition and analysis of data. LG contributed to analysis and interpretation of data, and drafted manuscript. All authors contributed to the article and approved the submitted version.

## Funding

This work was supported by the Natural Science Foundation of Shandong Province (2019GSF108273, 2017WS215, and ZR2018BH021), the Qingdao Outstanding Health Professional Development Fund, and the Qilu Health Leading Talent Project.

## Supplementary Material

The Supplementary Material for this article can be found online at: https://www.frontiersin.org/articles/10.3389/fonc.2021.693284/full#supplementary-material

## Conflict of Interest

The authors declare that the research was conducted in the absence of any commercial or financial relationships that could be construed as a potential conflict of interest.
