# Supplementary material for: Diagnostic Value of CircRNAs as Potential Biomarkers in Oral Squamous Cell Carcinoma: a Meta-Analysis
Source: Front Oncol. 2021 Jul 8;11:693284. doi: 10.3389/fonc.2021.693284 (PMC8295991; doi:10.3389/fonc.2021.693284)

Appendix 1

Search keywords for the systematic review:

• Circular RNA; circRNA; closed circular RNA; circular intronic RNA; ciRNA AND

• Oral neoplasm; oral cancer; oral carcinoma; oral tumor; oral squamous cell carcinoma; OSCC; oral cavity neoplasm; oral cavity tumor; oral cavity carcinoma; oral cavity cancer; oral cavity squamous cell carcinoma; OCSCC.

Appendix 2 Excluded full-text articles

Insufficient data

1. Ai Y, Tang Z, Zou C, Wei H, Wu S, Huang D. circ_SEPT9, a newly identified circular RNA, promotes oral squamous cell carcinoma progression through miR-1225/PKN2 axis. *Journal of Cellular and Molecular Medicine* (2020) 24(22):13266-77. doi: 10.1111/jcmm.15943.

2. Chen X, Yu J, Tian H, Shan Z, Liu W, Pan Z, et al. Circle RNA hsa:circRNA_100290 serves as a ceRNA for miR-378a to regulate oral squamous cell carcinoma cells growth via Glucose transporter-1 (GLUT1) and glycolysis. *Journal of Cellular Physiology* (2019) 234(11):19130-40. doi: 10.1002/jcp.28692.

3. Deng W, Fu J, Wang T, Chen JX, Fu LB, Peng W. Hsa_circRNA_101036 acts as tumor-suppressor in oral squamous cell carcinoma cells via inducing endoplasmic reticulum stress. *European Review for Medical and Pharmacological Sciences* (2020) 24(11):6111-21. PubMed PMID: WOS:000542190000031.

4. Dong W, Zhao L, Zhang S, Zhang S, Si H. Circ-KIAA0907 inhibits the progression of oral squamous cell carcinoma by regulating the miR-96-5p/UNC13C axis. *World J Surg Oncol* (2021) 19(1):75. Epub 2021/03/16. doi: 10.1186/s12957-021-02184-8. PubMed PMID: 33715625.

5. Gao L, Zhao CY, Li SM, Dou ZC, Wang QB, Liu JC, et al. circ-PKD2 inhibits carcinogenesis via the miR-204-3p/APC2 axis in oral squamous cell carcinoma. *Molecular Carcinogenesis* (2019) 58(10):1783-94. doi: 10.1002/mc.23065. PubMed PMID: WOS:000483693000006.

6. Meng Y, Zhao EY, Zhou Y, Qiang DX, Wang S, Shi L, et al. Circular RNA hsa_circ_0011946 promotes cell growth, migration, and invasion of oral squamous cell carcinoma by upregulating PCNA. *European Review for Medical and Pharmacological Sciences* (2020) 24(3):1226-32. doi: 10.26355/eurrev_202002_20175. PubMed PMID: WOS:000516575000027.

7. Qiu L, Zheng L, Gan C, Deng W, Sun Y, Wang T. circBICD2 targets miR-149-5p/IGF2BP1 axis to regulate oral squamous cell carcinoma progression. *Journal of Oral Pathology and Medicine* (2020). doi: 10.1111/jop.13156.

8. Saikishore R, Velmurugan P, Ranjithkumar D, Latha R, Sathiamoorthi T, Arun A, et al. The Circular RNA-miRNA Axis: A Special RNA Signature Regulatory Transcriptome as a Potential Biomarker for OSCC. *Molecular Therapy-Nucleic Acids* (2020) 22:352-61. doi: 10.1016/j.omtn.2020.09.001. PubMed PMID: WOS:000596958600001.

9. Shen Y, Su W, Yang H. Hsa-circ-005379 competitively binds tomiR-182-5p to mediate PTEN expression in oralsquamous cell carcinoma. *Cancer Research* (2020) 80(16 SUPPL). doi: 10.1158/1538-7445.AM2020-1395.

10. Shi D, Li H, Zhang J, Li Y. CircGDI2 Regulates the Proliferation, Migration, Invasion and Apoptosis of OSCC via miR-454-3p/FOXF2 Axis. *Cancer Manag Res* (2021) 13:1371-82. Epub 2021/02/20. doi: 10.2147/cmar.S277096. PubMed PMID: 33603482; PubMed Central PMCID: PMCPMC7886390.

11. Su W, Wang YF, Wang F, Yang HJ, Yang HY. Effect of circular RNA hsa_circ_0002203 on the proliferation, migration, invasion, and apoptosis of oral squamous cell carcinoma cells. *Hua xi kou qiang yi xue za zhi = Huaxi kouqiang yixue zazhi = West China journal of stomatology* (2019) 37(5):509-15. doi: 10.7518/hxkq.2019.05.011.

12. Wang L, Wei Y, Yan Y, Wang H, Yang J, Zheng Z, et al. CircDOCK1 suppresses cell apoptosis via inhibition of miR-196a-5p by targeting BIRC3 in OSCC. *Oncology Reports* (2018) 39(3):951-66. doi: 10.3892/or.2017.6174.

13. Yang H, Wang Y. Circular RNA expression in oral squamouscell carcinoma. *Cancer Research* (2020) 80(16 SUPPL). doi: 10.1158/1538-7445.AM2020-4814.

14. Zhang X, Cheng J, Liu S, Li R. Down-regulating circular RNA_0004674 delays the progression of oral squamous cell carcinoma through microRNA-377-3p/THBS1 axis. *Life sciences* (2021):119236. doi: 10.1016/j.lfs.2021.119236.

15. Zhu X, Du J, Gu Z. Circ-PVT1/miR-106a-5p/HK2 axis regulates cell growth, metastasis and glycolytic metabolism of oral squamous cell carcinoma. *Mol Cell Biochem* (2020) 474(1-2):147-58. Epub 2020/08/02. doi: 10.1007/s11010-020-03840-5. PubMed PMID: 32737775.

16. Zhu X, Shao P, Tang Y, Shu M, Hu WW, Zhang Y. hsa_circRNA_100533 regulates GNAS by sponging hsa_miR_933 to prevent oral squamous cell carcinoma. *J Cell Biochem* (2019) 120(11):19159-71. Epub 2019/07/13. doi: 10.1002/jcb.29245. PubMed PMID: 31297884.

No relevant results reported

1. Bi L, Zhang C, Yao Y, He Z. Circ-HIPK3 regulates YAP1 expression by sponging miR-381-3p to promote oral squamous cell carcinoma development. *Journal of Biosciences* (2021) 46(1). doi: 10.1007/s12038-021-00142-w.

2. Cai C, Zhi Y, Wang K, Zhang P, Ji Z, Xie C, et al. CircHIPK3 overexpression accelerates the proliferation and invasion of prostate cancer cells through regulating miRNA-338-3p. *Onco Targets Ther* (2019) 12:3363-72. Epub 2019/05/24. doi: 10.2147/ott.S196931. PubMed PMID: 31118688; PubMed Central PMCID: PMCPMC6503193.

3. Chen F, Li XH, Liu C, Zhang Y, Wang RJ. Circ-ABCB10 accelerates the malignant progression of oral squamous cell carcinoma by absorbing miRNA-145-5p. *Eur Rev Med Pharmacol Sci* (2020) 24(2):681-90. Epub 2020/02/06. doi: 10.26355/eurrev_202001_20045. PubMed PMID: 32016969.

4. Dou Z, Li S, Ren W, Wang Q, Liu J, Kong X, et al. Decreased expression of hsa_circ_0072387 as a valuable predictor for oral squamous cell carcinoma. *Oral Dis* (2019) 25(5):1302-8. Epub 2019/03/26. doi: 10.1111/odi.13094. PubMed PMID: 30908839.

5. Li JM, Tseng CW, Lin CC, Law CH, Chien YA, Kuo WH, et al. Upregulation of LGALS1 is associated with oral cancer metastasis. *Ther Adv Med Oncol* (2018) 10:1758835918794622. Epub 2018/08/31. doi: 10.1177/1758835918794622. PubMed PMID: 30159048; PubMed Central PMCID: PMCPMC6109855.

6. Palmieri A, Carinci F, Martinelli M, Pezzetti F, Girardi A, Cura F, et al. Role of the MIR146A polymorphism in the origin and progression of oral squamous cell carcinoma. *Eur J Oral Sci* (2014) 122(3):198-201. Epub 2014/03/13. doi: 10.1111/eos.12121. PubMed PMID: 24612133.

7. Qian C, Chen S, Li S, Wang Y, Yao J. Circ_0000003 regulates glutamine metabolism and tumor progression of tongue squamous cell carcinoma via the miR‑330‑3p/GLS axis. *Oncol Rep* (2021) 45(4). Epub 2021/03/03. doi: 10.3892/or.2021.7996. PubMed PMID: 33649795; PubMed Central PMCID: PMCPMC7934215.

8. Wang F, Wang YF, Su W, Yang HJ, Yang HY. [Effect of circular RNA hsa_circ_0063772 on proliferation, migration and invasion of oral squamous cell carcinoma cells]. *Zhonghua Kou Qiang Yi Xue Za Zhi* (2019) 54(8):561-7. Epub 2019/08/06. doi: 10.3760/cma.j.issn.1002-0098.2019.08.011. PubMed PMID: 31378036.

9. Wang J, Jiang C, Li N, Wang F, Xu Y, Shen Z, et al. The circEPSTI1/mir-942-5p/LTBP2 axis regulates the progression of OSCC in the background of OSF via EMT and the PI3K/Akt/mTOR pathway. *Cell Death Dis* (2020) 11(8):682. Epub 2020/08/23. doi: 10.1038/s41419-020-02851-w. PubMed PMID: 32826876; PubMed Central PMCID: PMCPMC7443145.

10. Wang J, Zhao SY, Ouyang SS, Huang ZK, Luo Q, Liao L. [Circular RNA circHIPK3 acts as the sponge of microRNA-124 to promote human oral squamous cell carcinoma cells proliferation]. *Zhonghua Kou Qiang Yi Xue Za Zhi* (2018) 53(8):546-51. Epub 2018/08/07. doi: 10.3760/cma.j.issn.1002-0098.2018.08.009. PubMed PMID: 30078268.

11. Yang Y, Ci HS, Mao YL, Li JW, Zuo JH. CircRNA_002178 promotes the proliferation and migration of oral squamous cell carcinoma cells by activating the Akt/mTOR pathway. *Eur Rev Med Pharmacol Sci* (2020) 24(11):6122-30. Epub 2020/06/24. doi: 10.26355/eurrev_202006_21507. PubMed PMID: 32572877.

12. Zhou HX, Wang LY, Chen S, Wang DD, Fang Z. [Effect of circular RNA hsa_circ_0008898 on oral squamous cell carcinoma and its mechanism]. *Zhonghua Kou Qiang Yi Xue Za Zhi* (2020) 55(8):578-85. Epub 2020/08/28. doi: 10.3760/cma.j.cn112144-20200109-00006. PubMed PMID: 32842350.

13. Deng W, Peng W, Wang T, Chen J, Qiu X, Fu L, et al. Microarray profile of circular RNAs identifies hsa_circRNA_102459 and hsa_circRNA_043621 as important regulators in oral squamous cell carcinoma. *Oncol Rep* (2019) 42(6):2738-49. Epub 2019/10/23. doi: 10.3892/or.2019.7369. PubMed PMID: 31638251.

14. Dou Z, Gao L, Ren W, Zhang H, Wang X, Li S, et al. CiRS-7 functions as a ceRNA of RAF-1/PIK3CD to promote metastatic progression of oral squamous cell carcinoma via MAPK/AKT signaling pathways. *Experimental Cell Research* (2020) 396(2). doi: 10.1016/j.yexcr.2020.112290.

15. Gao L, Wang QB, Zhi Y, Ren WH, Li SM, Zhao CY, et al. Down-regulation of hsa_circ_0092125 is related to the occurrence and development of oral squamous cell carcinoma. *International Journal of Oral and Maxillofacial Surgery* (2020) 49(3):292-7. doi: 10.1016/j.ijom.2019.07.014. PubMed PMID: WOS:000520949800002.

16. Han L, Cheng J, Li A. hsa_circ_0072387 Suppresses Proliferation, Metastasis, and Glycolysis of Oral Squamous Cell Carcinoma Cells by Downregulating miR-503-5p. *Cancer Biother Radiopharm* (2021) 36(1):84-94. Epub 2020/04/18. doi: 10.1089/cbr.2019.3371. PubMed PMID: 32302508.

17. Hao C, Wangzhou K, Liang Z, Liu C, Wang L, Gong L, et al. Circular RNA ITCH Suppresses Cell Proliferation but Induces Apoptosis in Oral Squamous Cell Carcinoma by Regulating miR-421/PDCD4 Axis. *Cancer Manag Res* (2020) 12:5651-8. Epub 2020/08/09. doi: 10.2147/cmar.S258887. PubMed PMID: 32765068; PubMed Central PMCID: PMCPMC7369364.

18. Liu J, Yang Q, Sun H, Wang X, Saiyin H, Zhang H. The circ-AMOTL1/ENO1 axis implicated in the tumorigenesis of OLP-associated oral squamous cell carcinoma. *Cancer Management and Research* (2020) 12:7219-30. doi: 10.2147/CMAR.S251348.

19. Liu JP, Jiang X, Zou AL, Mai ZY, Huang ZJ, Sun LY, et al. circIGHG-Induced Epithelial-to-Mesenchymal Transition Promotes Oral Squamous Cell Carcinoma Progression via miR-142-5p/IGF2BP3 Signaling. *Cancer Research* (2021) 81(2):344-55. doi: 10.1158/0008-5472.Can-20-0554. PubMed PMID: WOS:000611357000012.

20. Luo Y, Liu F, Guo J, Gui R. Upregulation of circ_0000199 in circulating exosomes is associated with survival outcome in OSCC. *Sci Rep* (2020) 10(1):13739. Epub 2020/08/15. doi: 10.1038/s41598-020-70747-y. PubMed PMID: 32792549; PubMed Central PMCID: PMCPMC7426867.

21. Ouyang SB, Wang J, Zhao SY, Zhang XH, Liao L. CircRNA_0109291 regulates cell growth and migration in oral squamous cell carcinoma and its clinical significance. *Iranian Journal of Basic Medical Sciences* (2018) 21(11):1186-91. doi: 10.22038/ijbms.2018.30347.7313. PubMed PMID: WOS:000445923100014.

22. Peng QS, Cheng YN, Zhang WB, Fan H, Mao QH, Xu P. circRNA_0000140 suppresses oral squamous cell carcinoma growth and metastasis by targeting miR-31 to inhibit Hippo signaling pathway. *Cell Death & Disease* (2020) 11(2). doi: 10.1038/s41419-020-2273-y. PubMed PMID: WOS:000543782600002.

23. Su W, Shen Y, Wang Y, Wang F, Hong X, Chen Y, et al. circPHIP promotes oral squamous cell carcinoma progression by sponging miR-142-5p and regulating PHIP and ACTN4 expression. *Molecular Therapy - Nucleic Acids* (2021) 23:185-99. doi: 10.1016/j.omtn.2020.10.038.

24. Su W, Sun S, Wang F, Shen Y, Yang H. Circular RNA hsa-circ-0055538 regulates the malignant biological behavior of oral squamous cell carcinoma through the p53/Bcl-2/caspase signaling pathway. *Journal of Translational Medicine* (2019) 17(1). doi: 10.1186/s12967-019-1830-6.

25. Su W, Wang YF, Wang F, Zhang BR, Zhang HY, Shen YH, et al. Circular RNA hsa_circ_0007059 indicates prognosis and influences malignant behavior via AKT/mTOR in oral squamous cell carcinoma. *Journal of Cellular Physiology* (2019) 234(9):15156-66. doi: 10.1002/jcp.28156. PubMed PMID: WOS:000470174200066.

26. Tan X, Zhou C, Liang Y, Lai Y. Circ_0001971 regulates oral squamous cell carcinoma progression and chemosensitivity by targeting miR-194/miR-204 in vitro and in vivo. *European Review for Medical and Pharmacological Sciences* (2020) 24(5):2470-81. doi: 10.26355/eurrev_202003_20515.

27. Wang J, Jiang CH, Li N, Wang F, Xu Y, Shen ZZ, et al. The circEPSTI1/mir-942-5p/LTBP2 axis regulates the progression of OSCC in the background of OSF via EMT and the PI3K/Akt/mTOR pathway. *Cell Death & Disease* (2020) 11(8). doi: 10.1038/s41419-020-02851-w. PubMed PMID: WOS:000568889800001.

28. Wang YF, Li BW, Sun S, Li X, Su W, Wang ZH, et al. Circular RNA Expression in Oral Squamous Cell Carcinoma. *Frontiers in Oncology* (2018) 8. doi: 10.3389/fonc.2018.00398. PubMed PMID: WOS:000446583100001.

29. Zhang Q, Jiang C, Ren W, Li S, Zheng J, Gao Y, et al. Circ-LRP6 mediates epithelial-mesenchymal transition and autophagy in oral squamous cell carcinomas. *Journal of Oral Pathology and Medicine* (2021). doi: 10.1111/jop.13163.

30. Zhang Y, Tang K, Chen L, Du M, Qu Z. Exosomal circgdi2 suppresses oral squamous cell carcinoma progression through the regulation of mir-424-5p/scai axis. *Cancer Management and Research* (2020) 12:7501-14. doi: 10.2147/CMAR.S255687.

31. Zhao W, Cui Y, Liu L, Qi X, Liu J, Ma S, et al. Splicing factor derived circular RNA circUHRF1 accelerates oral squamous cell carcinoma tumorigenesis via feedback loop. *Cell Death and Differentiation* (2020) 27(3):919-33. doi: 10.1038/s41418-019-0423-5.

32. Zheng Z, Ma X, Li H. Circular RNA circMDM2 accelerates the glycolysis of oral squamous cell carcinoma by targeting miR-532-3p/HK2. *Journal of Cellular and Molecular Medicine* (2020) 24(13):7531-7. doi: 10.1111/jcmm.15380.

33. Zhu LS, Wang YL, Li R, Xu XT, Li KY, Zuo CR. circ_BICD2 acts as a ceRNA to promote tumor progression and Warburg effect in oral squamous cell carcinoma by sponging miR-107 to enhance HK2. *American Journal of Translational Research* (2020) 12(7):3489-500. PubMed PMID: WOS:000558675900012.

Appendix 3 Risk of Bias and Applicability concerns in Individual Studies by QUADAS-2 tool.

| Item | Chen G 2020 | Dou Z 2019 | Fan C 2019 | Fan X 2021 | He T 2018 | Li B 2018 | Li L 2020 | Li X 2019 | Sun S  2018 | Sun W 2019 | Wang Z 2019 | Xia B 2019 | Zhang B 2020 | Yao W 2020 | Zhang H 2020 | Zhao S 2018 |
| --- | --- | --- | --- | --- | --- | --- | --- | --- | --- | --- | --- | --- | --- | --- | --- | --- |
| 1.Was a consecutive or random sample of patients enrolled? | U | U | U | N | U | U | U | U | U | U | U | N | U | U | U | U |
| 2.Was a case-control design avoided? | N | N | N | N | N | N | N | N | N | N | N | N | N | N | N | N |
| 3.Did the study avoid inappropriate exclusions? | N | N | N | N | N | N | N | N | N | N | N | N | N | N | N | N |
| 4.Were the index test results interpreted without knowledge of the results of the reference standard? | U | U | U | U | U | U | U | U | U | U | U | U | U | U | U | U |
| 1. If a threshold was used, was it pre-specified? | U | U | U | U | U | U | U | U | U | U | U | U | U | U | U | U |
| 1. Is the reference standards likely to correctly classify the target condition? | Y | Y | Y | Y | Y | Y | Y | Y | Y | Y | Y | Y | Y | Y | Y | Y |
| 1. Were the reference standard results interpreted without knowledge of the results of the index tests? | U | U | U | U | U | U | U | U | U | U | U | U | U | U | U | U |
| 1. Was there an appropriate interval between index test and reference standard? | Y | Y | Y | Y | Y | Y | Y | Y | Y | Y | Y | Y | Y | Y | Y | Y |
| 1. Did all patients receive the same reference standard? | Y | Y | Y | Y | Y | Y | Y | Y | Y | Y | Y | Y | Y | Y | Y | Y |
| 1. Were all patients included in the analysis? | Y | Y | Y | Y | Y | Y | Y | Y | Y | Y | Y | Y | Y | Y | Y | Y |

Appendix 4 meta-regression analysis outcome.

Appendix 5 TSA outcome


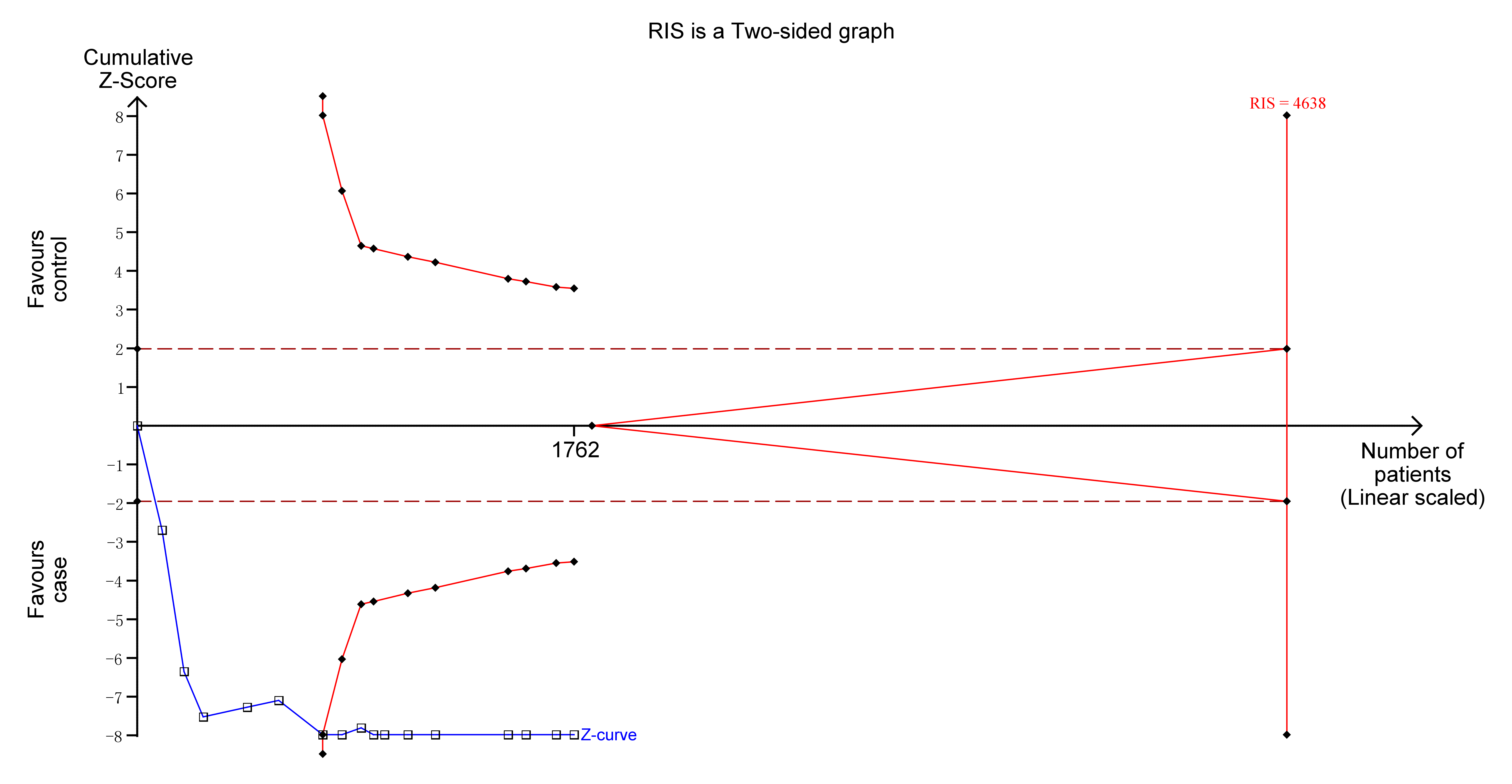

Supplement: Supplementary file 1 [file DataSheet_1.doc]
